# The ATP-dependent chromatin remodeling enzymes CHD6, CHD7, and CHD8 exhibit distinct nucleosome binding and remodeling activities

**DOI:** 10.1074/jbc.M117.779470

**Published:** 2017-05-21

**Authors:** Benjamin J. Manning, Timur Yusufzai

**Affiliations:** From the Department of Radiation Oncology, Dana-Farber Cancer Institute and Department of Biological Chemistry and Molecular Pharmacology, Harvard Medical School, Boston, Massachusetts 02215

**Keywords:** ATPase, chromatin remodeling, epigenetics, neurological disease, nucleosome

## Abstract

Proper chromatin regulation is central to genome function and maintenance. The group III chromodomain–helicase–DNA-binding (CHD) family of ATP-dependent chromatin remodeling enzymes, comprising CHD6, CHD7, CHD8, and CHD9, has well-documented roles in transcription regulation, impacting both organism development and disease etiology. These four enzymes are similar in their constituent domains, but they fill surprisingly non-redundant roles in the cell, with deficiencies in individual enzymes leading to dissimilar disease states such as CHARGE syndrome or autism spectrum disorders. The mechanisms explaining their divergent, non-overlapping functions are unclear. In this study, we performed an in-depth biochemical analysis of purified CHD6, CHD7, and CHD8 and discovered distinct differences in chromatin remodeling specificities and activities among them. We report that CHD6 and CHD7 both bind with high affinity to short linker DNA, whereas CHD8 requires longer DNA for binding. As a result, CHD8 slides nucleosomes into positions with more flanking linker DNA than CHD7. Moreover, we found that, although CHD7 and CHD8 slide nucleosomes, CHD6 disrupts nucleosomes in a distinct non-sliding manner. The different activities of these enzymes likely lead to differences in chromatin structure and, thereby, transcriptional control, at the enhancer and promoter loci where these enzymes bind. Overall, our work provides a mechanistic basis for both the non-redundant roles and the diverse mutant disease states of these enzymes *in vivo*.

## Introduction

**Figure 1.**
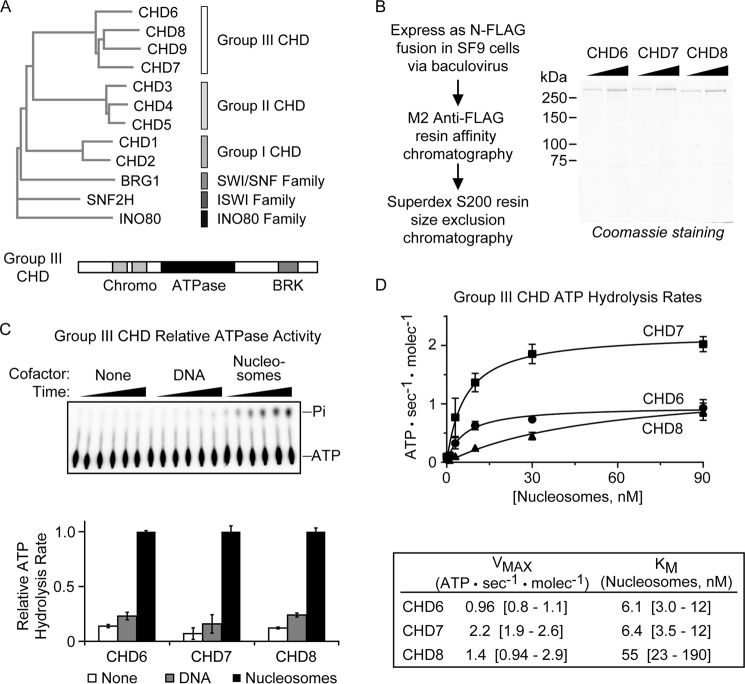
**Purification and ATP hydrolysis kinetics of group III CHD enzymes.** *A*, *top panel*, partial phylogenetic tree derived from sequence alignment of human ATP-dependent chromatin remodeling enzymes. *Bottom panel*, representative diagram of group III CHD enzymes. *B*, *left panel*, schematic for the CHD enzyme purification. *Right panel*, SDS-PAGE of 300 ng/600 ng of purified CHD enzymes. *C*, relative activation of the ATPase activity of CHD6, CHD7, and CHD8 either without substrate, with DNA substrate, or with mononucleosome substrate bearing 50-bp linker DNA on both sides of the nucleosome (the same DNA fragment as used in the DNA substrate reactions). *Top panel*, representative TLC autoradiogram from a time course experiment. *Pi*, inorganic phosphate. *Bottom panel*, quantification of substrate-specific ATP hydrolysis rates normalized to chromatin-dependent ATP hydrolysis. Values represent mean and S.D. (*n* = 3). *D*, *top panel*, quantification of nucleosome-stimulated ATP hydrolysis rates as a function of nucleosome concentration. *Bottom panel*, mean values were determined by Michaelis-Menten kinetics, and *brackets* denote the 95% confidence interval.

For eukaryotic cells, not all heritable information is encoded within the DNA sequence of their genomes. An additional level of information is present in the organization of the genome into chromatin, a nucleoprotein structure comprised at the lowest level as nucleosomes of DNA and histone proteins. Chromatin regulation is important for diverse cellular processes, from transcription and DNA repair to cell differentiation and organism development (1). Indeed, early screens in *Drosophila* looking for important developmental genes identified many chromatin-associated factors, including the ATP-dependent chromatin remodeling enzymes Brahma and Kismet (2). These enzymes are molecular motors that harness the energy from ATP hydrolysis to slide histone proteins along or off of DNA, thereby regulating the accessibility of the underlying DNA to various nuclear factors. In eukaryotes, there are several well-conserved families of ATP-dependent chromatin remodeling enzymes (Fig. 1*A*, *top panel*), distinguished from each other by their characteristic protein domains and biochemical activities (3–5).

One family of chromatin remodeling enzymes is the CHD^2^ family, defined by its eponymous tandem chromodomains, helicase domain, and DNA-binding domain. In humans, this family has nine members, classified into three groups according to domain similarity: group I (CHD1 and CHD2), group II (CHD3, CHD4, and CHD5), and group III (CHD6, CHD7, CHD8, and CHD9; Fig. 1*A*, *top panel*). The group III CHD enzymes are orthologs of the *Drosophila* Kismet enzyme (the only group III CHD in *Drosophila*) and are characterized by the Brahma and Kismet domains found at their C termini (Fig. 1*A*, *bottom panel*) (6). Although Kismet was initially identified as a member of the transcriptionally activating trithorax group of genes, the human orthologs of Kismet have been reported to act both as transcriptional coactivators and corepressors (7–13). Group III CHD enzymes are seen by ChIP analyses at the enhancer and promoter regions of genes, where they are thought to use their chromatin remodeling activities to regulate chromatin structure (14–17).

Despite their high degree of sequence identity (50–54%), the human group III CHD paralogs play non-redundant roles in the cell. Mutant versions of CHD7 and CHD8 lead to the distinct disease states of CHARGE syndrome (18, 19) and autism spectrum disorders (20–22), respectively, whereas little pathology is known for CHD6 (23, 24) and none for CHD9. One hypothesis explaining this phenotypic difference is that the paralogs interact with different subsets of proteins, such as sequence-specific transcription factors. An alternative hypothesis is that differences in temporal or cell type-specific expression of these paralogs could account for their different mutant phenotypes. A third and final hypothesis would be that, although these enzymes do share significant sequence identity, they might still exhibit distinct enzymatic properties.

In this report, we show that CHD6, CHD7, and CHD8 enzymes demonstrate different substrate specificities and remodeling activities. We find that CHD6 and CHD7 enzymes are capable of binding to short lengths of linker DNA, whereas the CHD8 enzyme requires longer DNA tracts. We also find that, consistent with this length requirement, CHD8 slides nucleosomes into positions that are flanked by longer linker DNA than CHD7-slid nucleosomes. Finally, although both CHD7 and CHD8 slide nucleosomes, we discover that CHD6 disrupts nucleosomes in a largely non-sliding manner. Thus, *in vivo*, gene promoters or enhancers bound to these different paralogs likely experience different chromatin remodeling activities that may synergize with or antagonize one another.

## Results

To biochemically characterize these enzymes, we first established a source of highly pure and active enzyme. Full-length CHD6, CHD7, and CHD8 cDNA were reverse-transcribed from HeLa cytoplasmic RNA and subcloned into the pFastBac vector along with an N-terminal FLAG tag. Following baculoviral expression in SF9 cells, the enzymes were enriched by anti-FLAG chromatography and then further purified by size-exclusion chromatography (Fig. 1*B*, *left panel*). The resulting CHD6, CHD7, and CHD8 enzymes were >95% pure, as judged by Coomassie staining on SDS-PAGE (Fig. 1*B*, *right panel*). CHD9 was also successfully subcloned, but we were unable to obtain a suitable amount of enzyme for this study.

We then sought to assess the activity of the purified enzyme preparations. We used a radiometric assay to quantitatively monitor the kinetics of ATP hydrolysis. Purified remodeling enzymes were incubated with ATP either in the absence of any cofactor or in the presence of a molar excess (60 nm) of DNA or nucleosomes. ATP hydrolysis was monitored over time (Fig. 1*C*, *top panel*), and the rates of ATP hydrolysis were calculated from the initial linear phase of the reaction. As shown previously for other CHD enzymes, CHD6, CHD7, and CHD8 are preferentially activated by nucleosomal substrates, showing 8- to 15-fold activation in the presence of chromatin over the absence of any cofactor (Fig. 1*C*, *bottom panel*). ATPase activity with DNA as a cofactor yields only a 2-fold activation over no cofactor at all, indicating the likely importance of histone contacts for stimulating ATPase activity, a feature known for other CHD family enzymes but not for SWI/SNF family enzymes (25).

Next, for each of these enzymes, we sought to quantify the ATPase activation by nucleosomes. ATP hydrolysis rates for each enzyme were measured over a 100-fold concentration range of nucleosomes, and fitting to Michaelis-Menten kinetics curves yielded *K_m_* and *V*_max_ values for ATP hydrolysis by each enzyme in response to nucleosomes (Fig. 1*D*, *top panel*). *V*_max_ data are within an approximate 2-fold window for all three enzymes, from one to two ATP hydrolyzed per second per enzyme, indicating the quality of enzyme preparation, activity, and standardization. Surprisingly, although CHD6 and CHD7 reach half-maximal ATPase activation at low nanomolar concentrations (6 nm) of nucleosome, CHD8 requires an almost 10-fold higher nucleosome concentration (55 nm) for similar activation (Fig. 1*D*, *bottom panel*).

**Figure 2.**
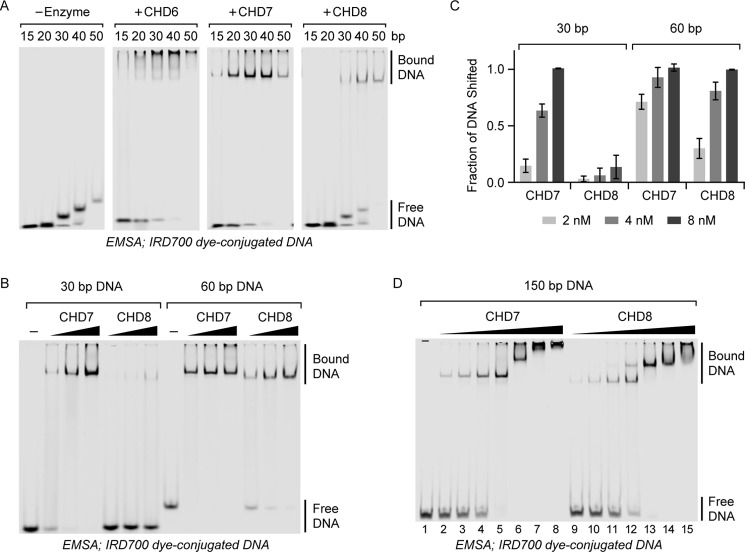
**DNA length sensitivities of group III CHD enzymes.** EMSAs were performed to investigate the binding of CHD6, CHD7, and CHD8 to dye-labeled DNA probes of various lengths. *A*, 2 nm DNA fragments were bound to 8 nm CHD6, CHD7, and CHD8. *B*, 2 nm either 30-bp (*left*) or 60-bp (*right*) DNA fragments were bound to 2, 4, or 8 nm CHD7 or CHD8 enzymes. *C*, quantification of *B*, expressed as a fraction of input signal present as enzyme-bound. Values are mean ± S.D. (*n* = 3). *D*, 2 nm 150-bp DNA fragment was bound to 0, 0.5, 1, 2, 4, 8, 16, or 32 nm CHD7 or CHD8 enzymes.

Prior work has highlighted the importance of enzyme–DNA contacts for enhancing the binding of CHD enzymes to nucleosomes (26). To determine whether differences in DNA binding affinities were the basis of the ATPase affinity discrepancy, we tested the ability of CHD6, CHD7, and CHD8 to bind DNA. IRDYE-labeled DNA fragments of varying length were incubated with an excess of CHD enzyme and visualized by native PAGE. Here, CHD6 and CHD7 are able to bind well to DNA fragments as small as 20 bp, whereas CHD8 does not bind well to DNA until the DNA reaches ∼40 bp in length (Fig. 2*A*). The DNA length sensitivity of CHD8 is consistent across multiple concentrations of CHD7 and CHD8 when binding 30- and 60-bp DNA fragments (Fig. 2*B*, quantified in Fig. 2*C*).

Of note, only a single species of enzyme–DNA complexes was seen with these ≤60-bp DNA fragments. When a longer, 150-bp DNA fragment is incubated with CHD7 and CHD8, two distinct migrating species are seen that likely represent the binding of one or two enzyme molecules to the DNA (Fig. 2*D*). These species occur at enzyme and DNA concentrations where shorter-length DNA fragments only display one single enzyme-bound DNA species. In the case of CHD7, a third, slower-migrating species is also observed (Fig. 2*D*, *lane 7*). These data are consistent with multiple CHD7 enzymes binding independently to different sites on the DNA rather than additional enzyme dimerizing with DNA-bound enzyme. However, minor cooperativity can be seen for CHD8, especially compared with CHD7 (Fig. 2*D*, *lanes 5* and *12*; 2 nm DNA, 4 nm enzyme), as inferred by the presence of double enzyme-bound DNA while unbound DNA is still available. Unfortunately, this analysis could not be performed with CHD6, whose enzyme–DNA complexes could not be resolved by EMSA (Fig. 2*A*).

**Figure 3.**
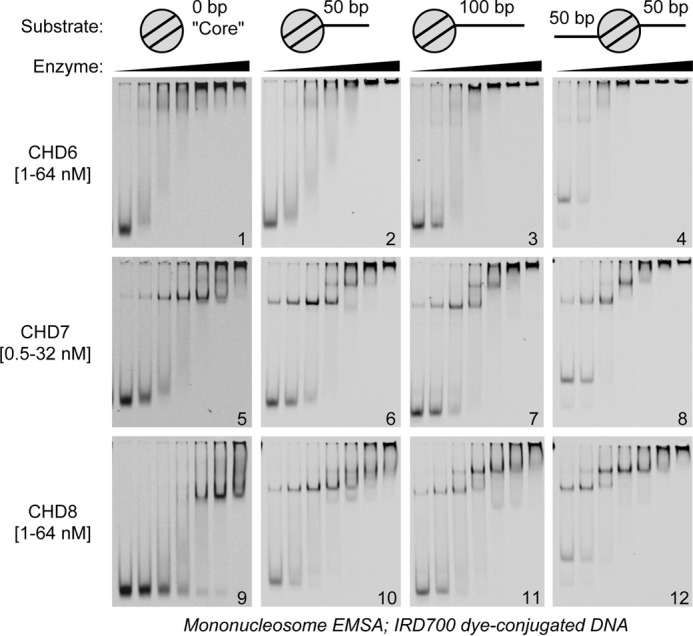
**Nucleosomal substrate specificities of group III CHD enzymes.** Dye-labeled chromatin substrates bearing different configurations of linker DNA (illustrated across the *top*) were bound to a 2-fold dilution series of CHD6, CHD7, or CHD8 (enzyme and concentration ranges are given at the *left*) and resolved and visualized by native PAGE.

The mean length of nucleosomal linker DNA differs from organism to organism and even between cell types within an organism (27, 28). We therefore asked how the DNA length sensitivities of CHD6, CHD7, and CHD8 correlate with how these enzymes bind to nucleosomes featuring different lengths of linker DNA. Mononucleosomes were assembled on IRDYE-labeled DNA with only 3–4 bp of DNA on either side of the nucleosome, with 50 bp of linker DNA on one end of the nucleosome, with 100 bp of linker DNA on one end of the nucleosome, or with 50 bp of linker DNA extending out of both ends of the nucleosome (Fig. 3, *top panel*; supplemental Fig. S1). Increasing amounts of the CHD enzymes were incubated with these substrates, and the reactions were resolved by native PAGE. CHD6 enzyme–nucleosome complexes again could not be visualized, but loss of the unbound nucleosome band occurred at similar rates for all substrates assayed, suggesting that CHD6 binding to nucleosomes could be largely linker-insensitive at these concentrations (Fig. 3, *gels 1–4*). Meanwhile, both CHD7- and CHD8-bound nucleosomes could be visualized. CHD7 binds well to nucleosomes in a largely linker-insensitive manner at these concentrations (Fig. 3, *gels 5–8*; supplemental Fig. S2*A*). In contrast, the binding of CHD8 to linker-free “core” mononucleosomes is detectably weaker in affinity than what was observed for CHD7 (Fig. 3, *gels 5* and *9*). This affinity difference is reduced by the presence of linker DNA (Fig. 3, *gels 9–12*; supplemental Fig. S2*B*).

A single enzyme–nucleosome complex band is visible for CHD7 and CHD8 when binding to core mononucleosomes; putative double enzyme-bound nucleosome bands are only visualized in the presence of linker DNA (Fig. 3, *gels 5* and *9 versus gels 6* and *10*). Consistent with CHD8 requiring 40+ bp of linker DNA, we observe more CHD8 double enzyme-bound complexes with 100-bp linker nucleosomes than with 50 bp linker nucleosomes, whereas, for CHD7, no such difference exists (Fig. 3, *gels 6* and *10 versus gels 7* and *11*). CHD8 binds with 2-fold higher affinity to the nucleosome with two 50-bp DNA linkers than to nucleosomes with only one 100-bp DNA linker (Fig. 3, *gel 11 versus gel 12*; supplemental Fig. S2*B*), emphasizing the contribution of both nucleosome and free DNA contacts to CHD8 binding. Indeed, the binding specificity of CHD8 correlates with the abilities of the different chromatin substrates to activate CHD8 ATPase activity (supplemental Fig. S3*A*). This behavior is distinct from what is known of ATPase regulation for chromatin-remodeling enzymes from the ISWI family (29). Finally, we again see double CHD8 enzyme-bound nucleosomes, even in the presence of unbound nucleosomes, which echoes the cooperativity of CHD8 binding seen in Fig. 2*C*. In summary, the preference of CHD8 for longer lengths of DNA manifests as a preference for nucleosomes with longer adjacent linker DNA, whereas CHD7 appears to bind equally well to nucleosomes with different amounts of or even no linker DNA.

**Figure 4.**
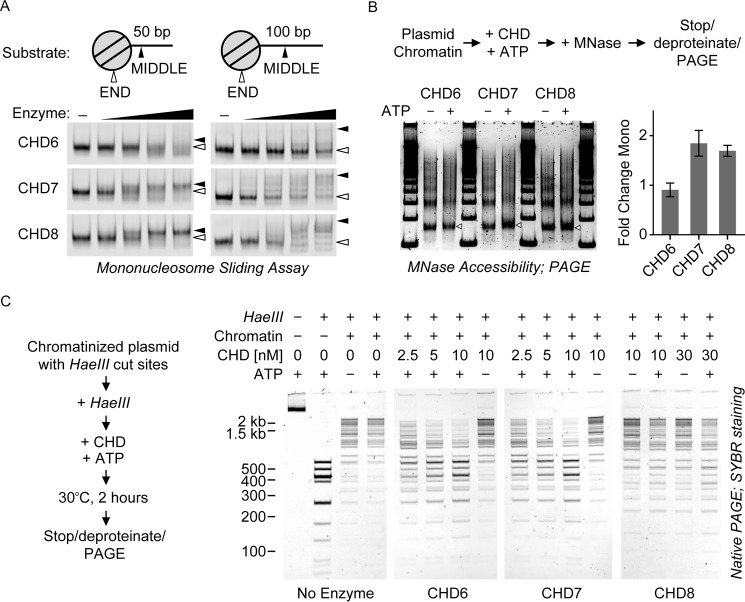
**Chromatin remodeling activities of group III CHD enzyme*s*.** *A*, mononucleosome sliding assay. 20 nm of dye-labeled, end-positioned nucleosome substrates (illustrated at the *top*) were incubated with 0, 0.25, 1, 4, or 16 nm CHD enzymes in the presence of ATP. After competing the enzymes off with excess plasmid DNA, the reaction products were visualized by native PAGE. The *white arrowheads* correspond to end-positioned species; the *black arrowheads* correspond to middle-positioned species. *B*, MNase accessibility assay. *Top panel*, reaction schematic; CHD enzymes were tested for their ability to alter the MNase accessibility of a chromatin array in an ATP-dependent manner. *Bottom left panel*, SYBR-stained native PAGE of MNase-freed product DNA species. The mononucleosome band is denoted by *white arrowheads. Bottom right panel*, the intensity of the mononucleosome band in each reaction was quantified as a fraction of the whole lane signal. Then, for each enzyme, the ratio of −ATP to + ATP mono band intensity was calculated. Values are mean ± S.D. (*n* = 3 or 5; see “Experimental Procedures”). *C*, HaeIII accessibility assay. *Left panel*, reaction schematic. CHD enzymes were tested for their ability to regulate HaeIII endonuclease accessibility of a chromatin array in an ATP-dependent manner. *Right panel*, SYBR-stained native PAGE of HaeIII-digested plasmid DNA species.

Based on the results of our binding studies, we then sought to correlate the binding properties of CHD6, CHD7, and CHD8 with their ATP-dependent chromatin remodeling activities. Some CHD enzymes have been reported to slide nucleosomes along DNA and to direct nucleosomal sliding toward adjacent free DNA (26, 30). To this end, we performed nucleosome sliding assays by incubating nucleosomes containing 50- or 100-bp linker DNA on one side (Fig. 4*A*, *top panel*) with increasing concentrations of enzyme. The reactions were then quenched with an excess of competitor unlabeled DNA and resolved via native PAGE. In this assay, nucleosome sliding will result in an enzyme-dependent shift of the nucleosomes from a higher-mobility position on the end of the DNA fragment (Fig. 4*A*, *white arrows*) toward a lower-mobility position in the middle of the DNA fragment (Fig. 4*A*, *black arrows*). CHD7 and CHD8 are able to slide nucleosomes containing 50-bp linker DNA (Fig. 4*A*, *left gels*). Indeed, ∼50% of nucleosomes are repositioned at a ratio of 1:20 enzyme to nucleosomes, corresponding to an approximate rate of 0.5 nucleosomes/min. CHD6, CHD7, and CHD8 also generate an unexplained high-mobility species at low enzyme concentrations that largely converts into the anticipated sliding product Fig. 4*A*, *black arrows*) at higher concentrations of CHD7 and CHD8. In contrast, CHD6 does not generate the anticipated sliding product of these nucleosomes, and the unexplained high-mobility species accumulates at higher concentrations of CHD6.

CHD7 and CHD8 enzymes are also capable of sliding 100-bp linker nucleosomes (Fig. 4*A*, *right gels*). Although the proportion of end-positioned nucleosomes decreases over time, there is notably less accumulation of a single, middle-positioned product. For CHD7, the repositioned nucleosomes appear to occupy a number of positions throughout the DNA, including the middle and the ends. This distribution correlates with the relative linker insensitivity of CHD7 binding. Meanwhile, CHD8 appears to slide nucleosomes to positions that have sufficient lengths of linker DNA on either side of the nucleosome. For nucleosomes positioned in the middle of the DNA fragment, CHD7 and CHD8 only modestly slide the nucleosomes (supplemental Fig. S3*B*). This finding is consistent with either the sliding reaction being in constant equilibrium between nucleosome position or with linker DNA of sufficient length being inhibitory for sliding. Finally, CHD6 again does not create high levels of anticipated sliding product; however, the end-positioned nucleosomes appear to decrease in intensity over time, signifying a conversion into an unexpected product (Fig. 4*A*). In contrast to CHD7 and CHD8, CHD6 is similarly robust at disrupting middle-positioned nucleosomes and end-positioned nucleosomes (supplemental Fig. S3*B*). No generation of free DNA species by CHD6 was observed in these reactions (supplemental Fig. S3*C*).

To study CHD6, CHD7, and CHD8 enzyme activities in the context of nucleosomal arrays, we asked whether these enzymes could increase the accessibility of array DNA to *trans* factors. We employed two remodeling assays to visualize these activities. First, we measured the ability of CHD6, CHD7, and CHD8 to alter the MNase accessibility of chromatin. We incubated chromatinized plasmid with the CHD enzymes in the presence or absence of ATP for 30 min and then performed a partial MNase digestion. The reactions were deproteinated, and the samples were resolved by PAGE and visualized by SYBR-safe staining. We found that CHD7 and CHD8 stimulated array MNase accessibility in an ATP-dependent manner, as visualized by the increased amount of liberated mononucleosome-length DNA (Fig. 4*B*, *arrowheads*; quantitation shown at the *right*), whereas CHD6 did not have this stimulatory effect.

Second, we assayed CHD6, CHD7, and CHD8 for their ability to stimulate restriction endonuclease accessibility (REA) of plasmid chromatin (Fig. 4*C*, *left panel*). The plasmid used in this assay is cleaved at 15 sites by the HaeIII endonuclease (Fig. 4*C*, No CHD panel, *lanes 1* + 2), but this cleavage is partially blocked by chromatin (Fig. 4*C*, *No Enzyme*, *third* and *fourth lanes*). The presence of an active chromatin-remodeling enzyme and ATP restores HaeIII accessibility. In striking contrast to the low sliding activity observed with the mononucleosome sliding assay, CHD6 showed robust remodeling activity in this REA assay, even at substoichiometric concentrations (2.5–10 nm enzyme *versus* 46 nm nucleosome). The CHD7 enzyme is similarly robust to CHD6. Finally, CHD8 is weak in this REA assay compared with its robust activity in the sliding assay and modestly enhances HaeIII cleavage even at almost 1:1 ratios of nucleosome to CHD8 (Fig. 4*C*, *CHD8*, *third* and *fourth lanes*).

## Discussion

**Figure 5.**
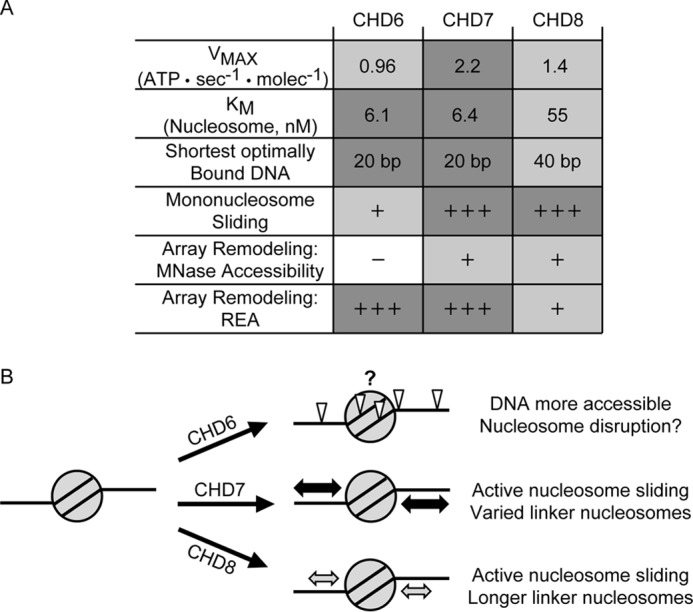
**CHD6, CHD7, and CHD8 exhibit distinct substrate specificities and remodeling activities.** *A*, table summarizing all of the specificities and enzymatic activities collected for the group III CHD enzymes throughout this manuscript. *B*, a model consistent with our data on the different activities of CHD6, CHD7, or CHD8 after being recruited to a hypothetical nucleosome.

In this report, we present our biochemical characterization of three group III CHD paralogs: CHD6, CHD7, and CHD8 (Fig. 5*A*) and the notable differences in binding and remodeling activities these enzymes exhibit. We find that all three enzymes display a similar specificity for nucleosomal substrates, and that they can hydrolyze ATP at similar maximal rates in response to nucleosomes. However, CHD8 has a 10-fold higher *K_m_* than either CHD6 or CHD7 despite binding equally well to this substrate by EMSA (Fig. 3, *gels 4*, *8*, and *12*). This observation correlates with our finding that CHD8 requires longer stretches of linker DNA to interact with compared with CHD6 or CHD7. This DNA length preference becomes emphasized in nucleosomal substrates, where CHD6 and CHD7 bind both to 155 bp core nucleosomes and linker-containing nucleosomes alike, whereas CHD8 binding is enhanced in the presence of linker DNA stretches. In terms of chromatin-remodeling activities, CHD7 and CHD8 are robust nucleosome sliding enzymes whereas CHD6 is not.

In the context of nucleosomal arrays, all three enzymes stimulate the accessibility of chromatin-obscured DNA to *trans* factors but in distinct manners. Both CHD7 and CHD8 are able to stimulate MNase liberation of mononucleosome-length DNA fragments from array molecules whereas CHD6 does not. In contrast, both CHD6 and CHD7 generate considerable chromatin array accessibility for the HaeIII restriction enzyme whereas CHD8 does not. The key difference of these array accessibility assays is the nuclease. Because MNase cleaves DNA in a largely sequence-independent manner, any increase in linker DNA accessibility would likely lead to a corresponding increase in MNase digestion. On the other hand, because HaeIII is a sequence-specific nuclease, small changes in the accessibility of linker DNA may not be sufficient to expose the full restriction site. For example, although an array of widely spaced nucleosomes may yield great potential for MNase to cleave linker DNA and liberate mononucleosomes, those widely spaced nucleosomes may never be slid far enough along DNA to reveal histone-occluded HaeIII sites. For CHD8, its ability to slide nucleosomes, to a limited subset of positions than observed for CHD7, could be sufficient to increase linker DNA accessibility to MNase but not to HaeIII. In contrast, an enzyme like CHD6 may disrupt but not space nucleosomes and might not change the mean accessibility of an array to limiting concentrations of MNase but, given sufficient time, could stimulate HaeIII accessibility of the nucleosomes it disrupts.

This study is the first systematic assessment and detailed biochemical comparison of the CHD6, CHD7, and CHD8 enzymes as well as the first *in vitro* assessment of the poorly studied CHD6 enzyme. Despite the lack of apparent sliding activity, CHD6 still disrupts nucleosome structure, and it dramatically increases the accessibility of chromatin arrays to the nuclease HaeIII. The chromatin disruption might occur through non-sliding mechanisms, such as through the stable generation of a nucleosome where histone-DNA contacts are disrupted. An earlier report from our laboratory found that CHD5, which lacks robust nucleosome sliding activity, is similarly robust at the REA assay because it catalyzes DNA unlooping from the nucleosome (31). An alternative hypothesis is that CHD6 catalyzes nucleosome accessibility via histone dimer displacement. Recent studies of the remodeling activities of *Saccharomyces cerevisiae* Chd1p from Levendosky *et al.* (32) make two key observations: first, that Chd1p can distinguish and remodel “hexasomes” (nucleosomes lacking one dimer of H2A–H2B), and second, that these hexasomes exhibit increased electrophoretic mobility relative to their parent nucleosomes. The second observation is consistent with our unexplained CHD6 sliding product being a hexasome. Although CHD6 may exhibit a still unknown remodeling activity, it is possible that CHD6 remodels nucleosomes by generating a stable hexasome, whereas CHD7 and CHD8 may generate such a high-mobility product only transiently. The difference between CHD6 and CHD7 and CHD8 is consistent with how CHD6 is the most evolutionarily divergent of the group III CHD paralogs.

CHD7 appears to be robust for nucleosome sliding and displays little preference for the linker DNA length of the resulting chromatin. Our data are consistent with prior biochemical studies on CHD7 (33) that had only looked at nucleosome sliding with 50-bp linker nucleosomes; our data also uncover differences in the relative linker length insensitivity between CHD7 and CHD8. As a result, CHD7 slides mononucleosomes to a variety of positions on DNA. In the context of a chromatin array, the sliding activity of CHD7 can strongly disrupt nucleosome–nucleosome spacing, stimulating MNase accessibility. In doing so, CHD7 is more competent at revealing nucleosomal DNA to *trans* factors such as HaeIII than CHD8 because CHD7 sliding is not bounded by a minimum linker DNA constraint. Consistent with our findings, CHD7 could act *in vivo* to foster the dynamic chromatin reorganization and accessibility of a locus during, for example, transcriptional activation.

In contrast, CHD8 prefers to establish chromatin with longer minimum lengths of linker DNA on either side of the nucleosome. *In vitro*, we see CHD8 stimulating MNase-dependent mononucleosomes release through its spacing and sliding activity, but this sliding is constrained by an increased requirement for DNA between nucleosomes. Moreover, although CHD7 and CHD8 do reposition middle-positioned nucleosomes by sliding, the repositioning is less dramatic than for end positioned-nucleosomes. We also report that CHD8 appears to hydrolyze ATP in a manner that correlates more with nucleosome binding affinity than with nucleosome sliding activity (Fig. 3, *gels 5–9*; supplemental Fig. S2, *A* and *B*). However, the ATPase activation *K_m_* values that we measure are lower in affinity than the apparent *K_D_* for nucleosome binding (Fig. 3). These discrepancies are likely due to the complex integration of DNA binding, nucleosome binding, ATP hydrolysis regulation, DNA translocation, and nucleosome sliding coupling that occur during chromatin remodeling. It is possible that, in the presence of a nucleosome with sufficient linker DNA, ATP hydrolysis occurs but is uncoupled from nucleosome sliding (34–36). Alternatively, the calf thymus octamers used here bear heterogeneous posttranslational modifications and may lack crucial modifications that are specifically read by these enzymes.

Our data for the role of the DNA length preference of CHD8 in directing nucleosome sliding is consistent with the data of other groups on CHD sliding regulation, which show yeast Chd1p positioning nucleosomes adjacent to key substrate cues *in vitro* and *in vivo* (37–39). Although uniform long-linker nucleosome positioning may increase DNA accessibility in some locus specific-contexts, on a larger scale, limiting linker length by creating regular wide-spaced nucleosomal arrays decreases DNA accessibility. This model is consistent with the established roles of CHD8 in transcriptional corepression and coactivation (8, 9, 12).

The disparate remodeling activities described here are consistent with the non-redundant phenotypes that result from mutation of the CHD6, CHD7, and CHD8 paralogs. It is likely that the chromatin landscape at any particular locus would vary based on which of these paralogs were recruited. ChIP sequencing datasets show that group III CHDs occupy important control loci, such as enhancers and gene promoters (14, 16, 17). Differential spacing or disruption of nucleosomes at such loci would reveal or occlude key binding sites for sequence-specific gene activators or the core transcriptional machinery. For example, it was reported that CHD7 is recruited during transcriptional activation of neural crest genes (10), whereas CHD8 functions as a corepressor at certain p53-controlled loci and Hox genes (8, 9, 40). As these paralogs exhibit different fundamental activities, one would expect different downstream phenotypes as a result of their misregulation. Moreover, our “different enzymatic activities” hypothesis for the distinct phenotypes of these paralogs is not mutually exclusive with alternative hypotheses, such as tissue/temporal expression control of specific paralogs or association of the paralogs with different sets of sequence-specific transcription factors. Indeed, group III CHD mutant phenotypes are likely a function of all three of these hypotheses.

## Experimental procedures

### Molecular cloning and protein bioinformatics

All primers were optimized/designed with Primer3Plus (http://www.bioinformatics.nl/cgi-bin/primer3plus/primer3plus.cgi/)^3^ (53) and ordered from IDT (standard purification, lowest nanomole scale); sequences are available upon request. One microgram of HeLa cytoplasmic RNA was reverse-transcribed into cDNA in a 10-μl reaction using the Invitrogen Superscript III first-strand synthesis system (Thermo Fisher, 18080093; variables as predicted from the product literature; primers for CHD6, CHD7, and CHD8 were kit-provided oligo(dT); CHD9, gene-specific primer). One-twentieth of the cDNA reaction was used as source material for ORF amplification by Phusion PCR in 20-μl reactions with HF buffer, except for CHD7, which required GC buffer + 3% DMSO (Thermo Fisher, F530S). The PCR product was purified with a PCR purification kit (Qiagen, 28104). For cloning into a standard N-terminal FLAG-tagging pFastBac vector (Life Technologies), 2 μg of either insert (Phusion primers containing appropriate restriction sites) or vector were digested with 20 units of NotI, XhoI, or SalI as appropriate (New England Biolabs; R0189S, R0146S, and R0138S; enzymes can be deduced from cut sites in cloning primers) in 1× New England Biolabs buffer 3.1 overnight at 37 °C. The digest product was purified by gel extraction (Qiagen, 28704) from a 0.7% agarose gel, as visualized on a white light table with crystal violet. Ligation was performed with the Roche Rapid DNA ligation kit according to the instructions (Sigma-Aldrich, 11635379001) and transformed into laboratory-made, standard XL-10 Gold *Escherichia coli* via the New England Biolabs XL-10 heat shock protocol. Successful clones were confirmed by restriction analysis and thoroughly sequenced (primer sequences are available upon request; Ensemble Consensus CDS number Uniprot entry number) to ensure complete sequence identity for full-length CHD6 (CCDS13317.1 Q8TD26), CHD7 (CCDS47865.1 Q3L8U1). The only mutation seen was the known CHD9 point mutant variant D2312E. Successful clones were integrated into a baculovirus bacmid by transformation into laboratory-made DH10BAC stocks via standard methods (see the Invitrogen protocol for the Bac-to-Bac® expression system). For phylogeny and sequence identity analysis, primary protein sequences (Uniprot entry numbers CHD3 Q12873, CHD4 Q14839, CHD1 O14646, CHD5 Q8TDI0, CHD2 O14647, INO80 Q9ULG1, SNF2H O60264, hBRM P51531, and BRG1 P51532; others given earlier) were aligned with Clustal Omega (http://www.ebi.ac.uk/Tools/msa/clustalo/) and visualized with ClustalW2 Simple Phylogeny (http://www.ebi.ac.uk/Tools/phylogeny/simple_phylogeny/).^3^

### Cell culture work

All insect cell (SF9) work was performed according to standard protocols (*e.g.* Ref. 41) in Grace's insect medium supplemented to 10% v/v with FBS and 1× with penicillin, streptomycin, and Amphotericin B. SF9 cells were grown in a 25 °C incubator; cells were grown attached to dishes until scale-up into 1 L spinner cultures (50 rpm). To generate P_0_ baculovirus, 80% confluent SF9 cells in 6-well plates were transfected with purified bacmid using Cellfectin® II (Thermo Fisher, 10362100) according to the protocol of the supplier. 200 μl of this P_0_ stock was used to inoculate 80% confluent SF9 cells in a 15-cm dish; after 3 days, the supernatant was harvested, clarified by centrifugation at 2000 × *g* for 5 min, and saved as virus P_1_ stock. 4 ml of P_1_ stock was used to inoculate 1 L of 1E6 cells per milliliter SF9 spinner cultures. After 3 days, cells were harvested by centrifugation at 1000 × *g* for 10 min at 4 °C. The pellet was resuspended in ice-cold 1× PBS + 200 μm PMSF to wash and then pelleted by centrifugation at 1000 × *g* for 10 min at 4 °C. The resulting pellet was flash-frozen in liquid N_2_ and stored at −80 °C until use. HeLa cells were grown according to standard protocols in Dulbecco's modified Eagle's medium supplemented to 10% v/v with FBS and 1× with penicillin and streptomycin. 1E7 mid-log phase HeLa cells were collected and used for cytoplasmic RNA extraction using the Qiagen RNeasy mini kit (Qiagen, 74104; supplier protocol).

### Protein expression and purification

The SF9 pellet was rapidly thawed in 25 °C water and then transferred immediately onto ice when thawing was apparent. The pellet was wet and resuspended in 16 ml buffer A (20% glycerol, 20 mm sodium Hepes (pH 7.65), 500 mm NaCl, 1.5 mm MgCl_2_, 200 μm EDTA, 0.01% v/v Nonidet P-40, 1 mm DTT, 200 μm PMSF, 500 μm benzamidine, 1 μg/ml aprotinin, 1 μg/ml pepstatin A, and 1 μg/ml leupeptin). Cells were lysed in a 40-ml Dounce homogenizer using a tight pestle, on ice in a 4 °C room, performing 40 strokes over 30 min. Lysis was monitored microscopically. Lysed cells were transferred into Oak Ridge-style centrifuge tubes and pooled with an 8-ml wash of buffer A to collect remaining lysate from the homogenizer. Samples were spun at 53,200 × *g* at 4 °C for 20 min, the clarified supernatant was transferred to a new tube, and the spin was repeated. The supernatant from the second spin was transferred to a 50-ml conical vial, mixed with 300 μl of 50% M2 anti-FLAG resin slurry (Sigma, A2220–5ML; the resin was prewashed at 4 °C once in 1 ml of PBS, twice in 1 ml of 0.1 m glycine HCl (pH 2.8), and then three times in 1 ml of buffer A; all washes were done in batch mode to sufficiently resuspend the resin, all spins: 900 × *g*, 4 °C, 30 s) and incubated for 3 h at 4 °C on a rotator (∼30 rpm). Resin was collected, washed with 25 ml of buffer A twice, and then washed with 10 ml of buffer B (same as buffer A, but [NaCl] = 150 mm). After transfer into a 1.5 ml Eppendorf tube, the resin was washed five more times with 1 ml of buffer B, and then sample was eluted with 150 μl of buffer C (buffer B with 0.2 mg/ml FLAG peptide; Sigma, F3290) three times for 15 min. The pooled eluate was mixed and centrifuged at 14,000 × *g* at 4 °C for 10 min, and the supernatant was mixed with an equal volume of buffer D (same as buffer B but without glycerol). The sample was subjected to gel filtration chromatography through a Superdex 200 HiLoad 16/600 column on a GE Healthcare ÄKTAPurifier in buffer E (same as buffer B but with 10% v/v glycerol and lacking aprotinin, pepstatin, and leupeptin). 1-ml fractions were taken; fractions containing pure CHD enzyme were identified through SDS-PAGE and Coomassie staining, pooled, and concentrated to 300–600 μl (Millipore, UFC901008). A single peak fraction occurred at ∼47 ml for all enzymes. Concentrated enzyme was mixed and centrifuged at 14,000 × *g* 4 °C for 10 min, and the supernatant was aliquoted and flash-frozen in N_2_(L). Enzyme concentrations were determined through a combination of Bradford assay (Bio-Rad, 5000001), ImageJ (https://imagej.nih.gov/ij/) densitometry (three-point protein gradient, SDS-PAGE, and Coomassie staining) of CHD enzymes against each other as well as against ovalbumin standard (42) and UV absorbance spectroscopy (43, 44). Essential biochemical parameters were confirmed by assay repeats with separate purifications of the same enzyme. Average yield was ∼40 μg of highly purified protein (150–275 nm enzyme, 300–600 μl) per liter of SF9 culture.

### Nucleic acid and chromatin preparation

The supercoiled pGIE-0 plasmid (54) was amplified in DH5α *E. coli* and purified by Maxiprep (Qiagen, 12662). 601 nucleosome positioning sequence (NPS)-containing DNA fragments were amplified by PCR from the plasmid (Addgene, 26656 (45)) by Phusion PCR (Thermo Fisher, F530S; primer sequences are available upon request; variables as predicted from product literature) and subsequently purified with a PCR purification kit (Qiagen, 28104). DNA fragments used in the experiments shown in Fig. 2 were constructed by annealing a 601 sequence-derived, IR700dye-labeled oligonucleotide (46) with a complementary oligonucleotide. Briefly, complementary oligonucleotides were mixed at 45 μm each in 10 mm Tris (pH 8.0), 50 mm NaCl, 1 mm EDTA (50-μl reactions), heated to 95 °C in a thermocycler, ramp-cooled to 25 °C over 70 min, and then stored at 4 °C. All DNA concentrations were determined by nanodrop spectrophotometry and confirmed by stoichiometric incorporation into mononucleosomes with the same histone octamer. Calf thymus histone protein powder (Worthington, 31D12695) was dissolved in guanidine-containing unfolding buffer, refolded into histone octamers by dialysis into 1 × Tris-EDTA (TE) plus 2 m NaCl, and the stoichiometric histone octamer was purified by size-exclusion chromatography as described previously for recombinant histone proteins (47). To reconstitute chromatin, DNA and histone octamers were stoichiometrically mixed in TE plus 2 m NaCl and then assembled by stepwise salt dialysis into TE plus 1 m/0.8 m/0.6 m/50 mm NaCl as described previously (48) in the dark so as not to bleach the IRDYE. Varying ratios of nucleosome to DNA fragment (0.8 to 1.2) were reconstituted for all constructs; the optimally saturated reconstitutions were chosen based on native PAGE as described previously (49) and LICOR scanner-visualized, looking for the reconstitution with the brightest nucleosomal signal and the highest ratio of nucleosome to free DNA signal (see *stars* in supplemental Fig. S1).

### ATP hydrolysis assays

ATPase assays were performed as described previously (33, 46). 2.5–10 nm (final concentration) purified CHD enzyme was mixed with varying concentrations of substrate on ice in ATPase buffer (20 mm Tris HCl (pH 8.0), 50 mm NaCl, 5 mm MgCl_2_, 5% v/v glycerol, 200 μm DTT, 0.1% v/v Tween 20, and 100 μg/ml BSA). Substrates were either mononucleosomes with 50-bp linker DNA on both sides or the DNA used for reconstituting such mononucleosomes. Reactions were moved to a 30 °C heat block, and, at time 0, a mix of trace [δ-^32^P]ATP and excess cold ATP was added to a final concentration of 250 μm. Time points (1 μl) were taken and stopped by spotting onto PEI Cellulose thin-layer chromatography plates that had been prerun in Milli-Q deionized H_2_O and dried. Post-experiment, the plates were developed in a chamber equilibrated with 4.6% formic acid and 0.5 m LiCl. Plates were dried, exposed to storage phosphor screens for 3–6 h, and scanned on a Bio-Rad Personal Molecular Imager. Quantification was performed using Bio-Rad Image Lab v. 4.0 software, the fraction of ATP hydrolyzed being calculated from the fraction of total lane radio signal that was migrating in the topmost band. Rates of ATP hydrolysis were calculated from the slopes of time point ranges with linear hydrolysis curves. Experiments were conducted in triplicate; *error bars* represent standard deviation. *V*_max_ and *K_m_* parameters (with their 95% confidence intervals) were calculated by fit to Michaelis-Menten kinetics using Prism v. 7 (GraphPad).

### Substrate affinity assays

0–128 nm (concentration varied by experiment) purified CHD enzyme in EMSA buffer (20 mm Tris HCl (pH 8.0), 75 mm NaCl, 5 mm MgCl_2_, 10% v/v glycerol, 1 mm DTT, 0.1% v/v Tween 20, and 100 μg/ml BSA) was mixed with an equal volume of 4 nm IRDYE-conjugated substrate molecule in EMSA buffer. Reactions proceeded for 30 min at 25 °C. Samples were directly loaded into a 0.5× TBE 4% (29:1) native PAGE and electrophoresed at 100 V for 40 min to 1 h, depending on the substrate molecule. Gels were visualized by scanning on a LICOR imager while still between the glass plates. Experiments were repeated in triplicate; the representative gels shown were selected from experimental repeats. For quantitation, the signal of the enzyme-bound band was measured relative to the input unbound substrate signal. Repeat mean ± S.D. are graphed.

### Sliding assay

Sliding assays were performed mostly as described previously (33, 50, 51). 40 nm IRDYE-conjugated mononucleosome in EMSA buffer plus 2 mm ATP was mixed with an equal volume of 0–32 nm (concentration varies by experiment) purified CHD enzyme in EMSA buffer on ice. At time 0, reactions were transferred to a 30 °C water bath, and the reactions proceeded for 20 min. Reactions were stopped by transfer into an ice bucket and addition of 2.5 μl of QUENCH (20 mm Tris (pH 8.0), 50 mm NaCl, 1 mg/ml unlabeled supercoiled plasmid DNA, 50 mm EDTA, and 10% v/v glycerol) to a 10-μl reaction volume. Samples then sat at 25 °C for 10 min prior to native PAGE and LICOR scanning as described above. Experiments were repeated in triplicate; the representative gels shown were selected from experimental repeats.

### MNase accessibility assay

200 ng of chromatinized pGIE-0 plasmid in MNase buffer (20 mm Tris (pH 8.0), 50 mm NaCl, 5 mm MgCl_2_, 5% v/v glycerol, 1 mm DTT, 0.1% v/v Tween 20, and 1 mm ATP) was mixed with purified CHD enzyme in MNase buffer on ice, and then the reaction (10 μl) was shifted to 30 °C. After 30 min, 1.5 mm final CaCl_2_ and 1 gel unit of MNase (New England Biolabs, M0247S) were supplemented, and the reaction proceeded at 37 °C for 10 min. Reactions were shifted onto ice, stopped by addition of 1.1 μl of 110 mm EDTA (pH 8.0), and deproteinated by phenol/chloroform extraction. DNA was immediately mixed with loading dye and subjected to 0.5× TBE 4% (29:1) native PAGE at 100 V for 40 min. The gel was stained with a 1:10,000 dilution of SYBR Safe (Thermo, S33102) in 0.5× TBE and visualized in a Bio-Rad XR using Bio-Rad Image Lab v. 4.0 software. Experiments were repeated in triplicate (except CHD7, where *n* = 5); the representative gel shown was selected from experimental repeats. The mononucleosome band signal was measured as a percentage of the whole lane signal, and a ratio of mononucleosome band intensity with ATP to without ATP was calculated. Repeat mean ± S.D. are graphed.

### HaeIII accessibility assay

Accessibility assays were performed as described previously (46, 52). 100 ng of chromatinized pGIE-0 plasmid (∼46 nm nucleosome, final concentration) and 25 units of HaeIII restriction enzyme (New England Biolabs, R0108T) were mixed with 0–30 nm (concentration varied by experiment) purified CHD enzyme in in 1× New England Biolabs CutSmart buffer (20 mm Tris acetate (pH 7.9) at 25 °C, 50 mm potassium acetate, 10 mm magnesium acetate, and 100 μg/ml BSA; New England Biolabs, B7204S) on ice (with or without 3 mm ATP, final concentration). At time 0, the reaction (20 μl) was shifted to 30 °C. After 2 h, the reaction was stopped by addition of 125 μl of STOP solution (1% w/v SDS, 200 mm NaCl, 250 μg/ml glycogen, 20 mm EDTA (pH 8.0)) and 3 μl of 2.5 mg/ml Proteinase K solution. Digestion proceeded at 42 °C for 5 min, after which samples were extracted with an equal volume of phenol/chloroform, and the resultant aqueous phase was precipitated with 425 μl of ethanol. The nucleic acid pellet was resuspended in 6 μl of 1× gel loading dye and subjected to 0.5× TBE 4% (29:1) native PAGE at 100 V for 40 min. The gel was SYBR-stained and visualized as above. Experiments were repeated in triplicate; the representative gels shown were selected from experimental repeats.

## Author contributions

T. Y. and B. J. M. initiated the project. B. J. M. conducted the experiments. T. Y. and B. J. M. analyzed the data and wrote the manuscript.

## Supplementary Material

Supplemental Data

## Footnotes

3 Please note that the JBC is not responsible for the long-term archiving and maintenance of this site or any other third party-hosted site.

## Footnotes

2 The abbreviations used are:

CHD: chromodomain–helicase–DNA-binding
REA: restriction endonuclease accessibility
TBE: Tris borate-EDTA
TE: Tris-EDTA.
