# Supplementary material for: The ATP-dependent chromatin remodeling enzymes CHD6, CHD7, and CHD8 exhibit distinct nucleosome binding and remodeling activities
Source: J Biol Chem. 2017 May 21;292(28):11927–36. doi: 10.1074/jbc.M117.779470 (PMC5512084; doi:10.1074/jbc.M117.779470)
Supplement: Supplemental Data [file supp_292_28_11927__index.html]

The ATP-dependent Chromatin Remodeling Enzymes CHD6, CHD7, and CHD8 Exhibit Distinct Nucleosome Binding and Remodeling Activities — The ATP-dependent Chromatin Remodeling Enzymes CHD6, CHD7, and CHD8 Exhibit Distinct Nucleosome Binding and Remodeling Activities — The ATP-dependent chromatin remodeling enzymes CHD6, CHD7, and CHD8 exhibit distinct nucleosome binding and remodeling activities — CHD6, CHD7, and CHD8 nucleosome binding and remodeling — Supplemental Data 

# The ATP-dependent chromatin remodeling enzymes CHD6, CHD7, and CHD8 exhibit distinct nucleosome binding and remodeling activities

## Supplemental Data

- Supplemental Figure Legends (.docx, 14 KB) - Supplemental Figure Legends
- Supplemental Figure 1 (.pdf, 114 KB) - Supplemental Figure 1
- Supplemental Figure 2 (.pdf, 46 KB) - Supplemental Figure 2
- Supplemental Figure 3 (.pdf, 98 KB) - Supplemental Figure 3
