# Supplementary material for: The ATP-dependent chromatin remodeling enzymes CHD6, CHD7, and CHD8 exhibit distinct nucleosome binding and remodeling activities
Source: J Biol Chem. 2017 May 21;292(28):11927–36. doi: 10.1074/jbc.M117.779470 (PMC5512084; doi:10.1074/jbc.M117.779470)
Supplement: Supplemental Data [file 10.1074_M117.779470_jbc.M117.779470-1.docx]

**Supplemental Figure Legends**

Figure S1. *Mononucleosome Substrates Used in this Study.* 100 ng DNA equivalents of chromatin, reconstituted using increasing amounts of octamer on the indicated IRDYE-labeled DNA substrates, were resolved by Native PAGE and visualized with a fluorescent scanner. Stars indicate mononucleosomes of ideal saturation that were used in sliding assays.

Figure S2. *Quantification of CHD7 and CHD8 Nucleosome EMSA.* Quantification of CHD7 (A) and CHD8 (B) nucleosome EMSAs, as seen in Figure 3. Binding is expressed as the fraction of input nucleosome signal that is present in the enzyme-bound bands. Values are mean and SD [n=3].

Figure S3. *Supplementary Data on the Nucleosome Specificities for CHD6, CHD7 and CHD8.* (A) Activation of the ATPase activity (250 µM total ATP) of CHD8 (10 nM) with the indicated mononucleosome substrates (30 nM). Bars represent mean and SD [n=3]. (B) Top: 2 nM, 5 nM, 10 nM and 20 nM CHD8 mobilizes both end-positioned (left) and middle-positioned (right) nucleosomes by sliding assay. Light and dark arrows indicate nucleosome position as in Figure 4A. Bottom: same as Top, but with 10 nM or 20 nM CHD6 or CHD7. (C) ATP-dependent chromatin remodeling of end-positioned nucleosomes by CHD6 does not result in the release of free DNA (free DNA electrophoreses at the indicator circle), performed as a standard sliding assay. Light and dark arrows indicate nucleosome position as in Figure 4A.
