# Supplementary figures and images for: The ATP-dependent chromatin remodeling enzymes CHD6, CHD7, and CHD8 exhibit distinct nucleosome binding and remodeling activities
Source: J Biol Chem. 2017 May 21;292(28):11927–36. doi: 10.1074/jbc.M117.779470 (PMC5512084; doi:10.1074/jbc.M117.779470)

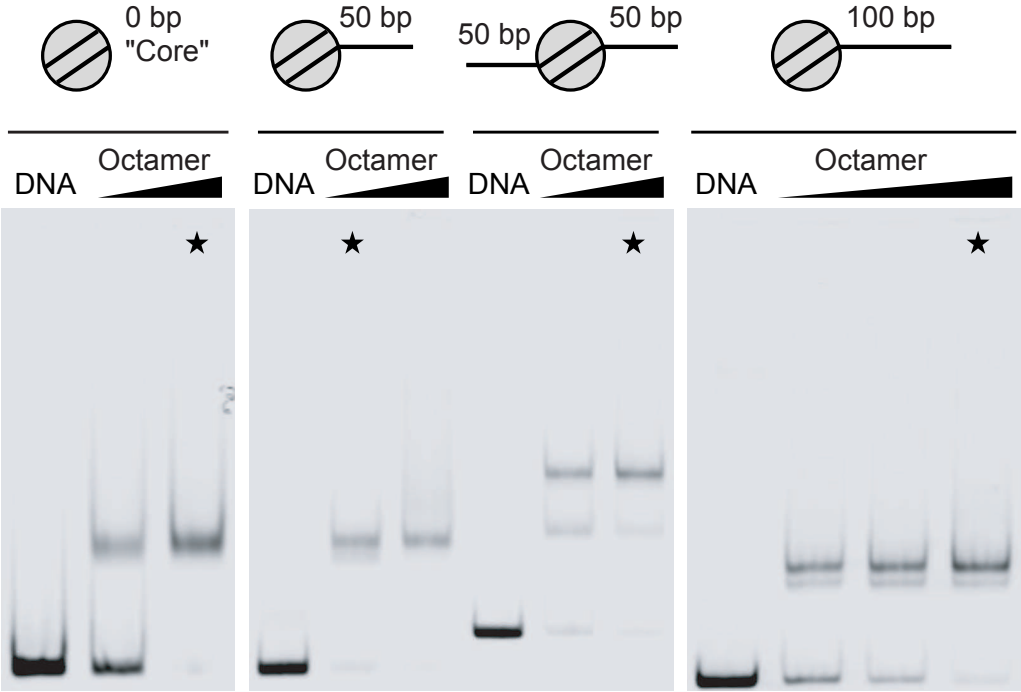

*Mononucleosome Reconstitution*

Supplement: Supplemental Data [file 10.1074_M117.779470_jbc.M117.779470-2.pdf]

A

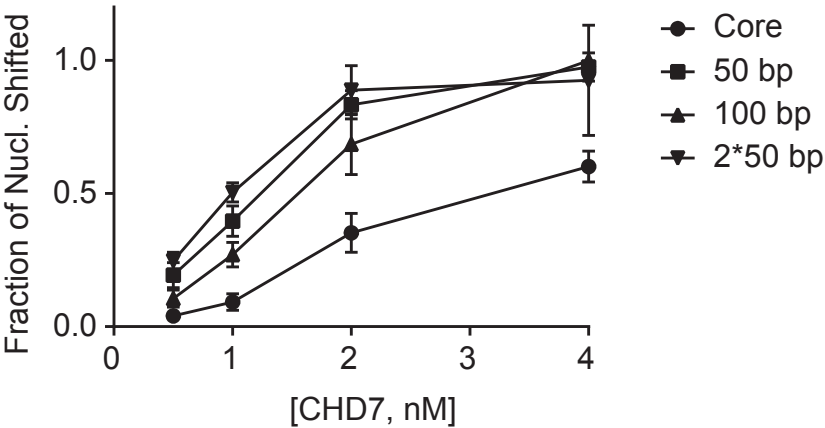

B

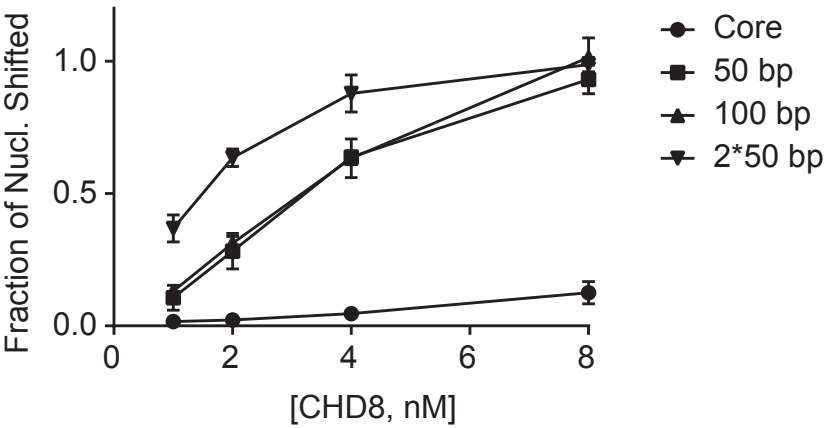

Supplement: Supplemental Data [file 10.1074_M117.779470_jbc.M117.779470-3.pdf]

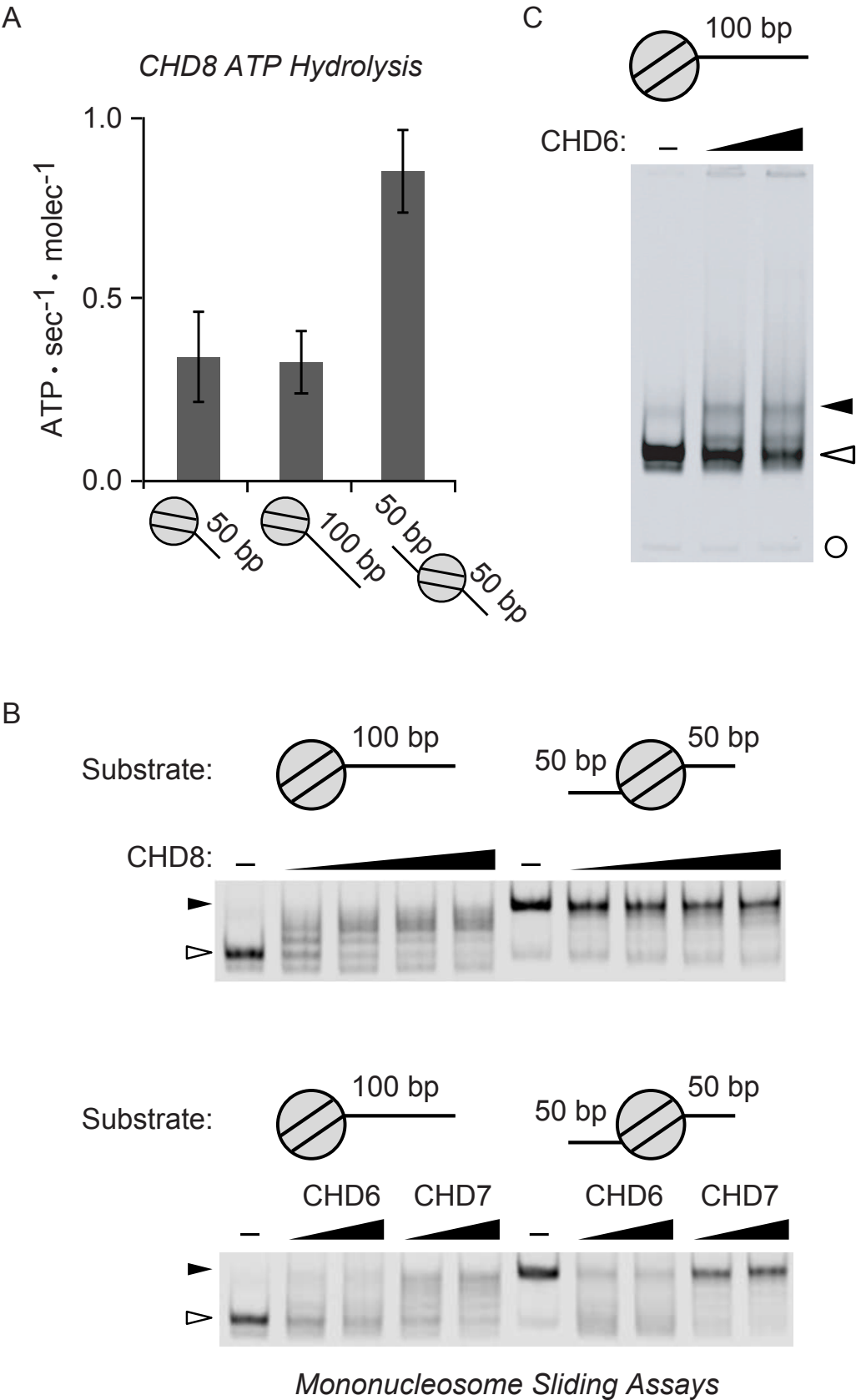

Supplement: Supplemental Data [file 10.1074_M117.779470_jbc.M117.779470-4.pdf]
